# LC3-Associated Phagocytosis (LAP): A Potentially Influential Mediator of Efferocytosis-Related Tumor Progression and Aggressiveness

**DOI:** 10.3389/fonc.2020.01298

**Published:** 2020-08-05

**Authors:** Patrick F. Asare, Eugene Roscioli, Plinio R. Hurtado, Hai B. Tran, Chui Yan Mah, Sandra Hodge

**Affiliations:** ^1^Department of Medicine, University of Adelaide, Adelaide, SA, Australia; ^2^Department of Thoracic Medicine, Royal Adelaide Hospital, Adelaide, SA, Australia; ^3^Department of Renal Medicine, Royal Adelaide Hospital, Adelaide, SA, Australia; ^4^South Australian Health and Medical Research Institute, Adelaide, SA, Australia; ^5^Freemasons Foundation Centre for Men's Health, Adelaide, SA, Australia

**Keywords:** efferocytosis, tumor cell apoptosis, LAP, tumor immune response, M2 macrophage activation

## Abstract

One aim of cancer therapies is to induce apoptosis of tumor cells. Efficient removal of the apoptotic cells requires coordinated efforts between the processes of efferocytosis and LC3-associated phagocytosis (LAP). However, this activity has also been shown to produce anti-inflammatory and immunosuppressive signals that can be utilized by live tumor cells to evade immune defense mechanisms, resulting in tumor progression and aggressiveness. In the absence of LAP, mice exhibit suppressed tumor growth during efferocytosis, while LAP-sufficient mice show enhanced tumor progression. Little is known about how LAP or its regulators directly affect efferocytosis, tumor growth and treatment responses, and identifying the mechanisms involved has the potential to lead to the discovery of novel approaches to target cancer cells. Also incompletely understood is the direct effect of apoptotic cancer cells on LAP. This is particularly important as induction of apoptosis by current cytotoxic cancer therapies can potentially stimulate LAP following efferocytosis. Herein, we highlight the current understanding of the role of LAP and its relationship with efferocytosis in the tumor microenvironment with a view to presenting novel therapeutic strategies.

## Introduction

Induction of apoptotic cell death in tumors serves to promote the body's main defense against cancer. Therapeutic intervention that promotes the apoptotic death of tumor cells restricts their growth, proliferation and survival and is a frontline strategy in anti-tumor therapy. However, apoptotic cells can directly (and indirectly) influence tumor progression and survival. Studies suggest that these effects are due to cellular events that are called in to remove dead or dying cells from the tumor microenvironment. A significant consequence of these events is the production of anti-inflammatory and immunosuppressive signals that then can facilitate the growth and progression of tumor cells (1, 2). Therefore, a better understanding of the processes that lead to the clearance of apoptotic cells may provide critical clues to help prevent the potential pro-tumor actions of dead or dying cells. Here we discuss two such cellular events; efferocytosis and LC3 Associated Phagocytosis (LAP).

Efferocytosis is a term coined by Peter Henson and colleagues in 2003 to describe the engulfment and clearance of cells undergoing apoptosis by phagocytes (3). Defects in efferocytosis can result in the rupture of uncleared apoptotic bodies and the subsequent leakage of cytosolic contents onto adjacent cells and exposure of tissues to oxidants, harmful enzymes and factors including caspases and other proteases (4, 5). This can induce inflammation and tissue damage through a process referred to as secondary necrosis (6, 7). Less understood is that during efferocytosis, signals from neighboring cells can initiate particular components of canonical autophagy to engage with the phagosome to increase the efficacy of the digestion of engulfed cellular cargo (8, 9). When components of autophagy are recruited to cargo-containing single membrane phagosomes to form LAPosome for digestion and clearance, the process is called LAP. Hence, LAP is a form of non-canonical autophagy that represents a hybrid between the processes governing the autophagic clearance of intracellular factors and those that accept extracellular cargos for degradation.

As the name suggests, a distinguishing feature of LAP is the recruitment of microtubule-associated protein light chain 3 (LC3) to the phagosome membrane after internalization of apoptotic cells or pathogens (10). In the normal situation, LAP is vital to resolve inflammation, promote wound healing and prevent auto-immunity and immune-mediated tissue damages (11, 12). Reports by Martinez et al. show that dysregulation of LAP impairs efferocytosis, culminating in necrosis of the phagocyte that has engulfed the uncleared cell and uncontrolled inflammation (12). These findings suggest that LAP may be essential for efferocytosis and preventing the many deleterious consequences of secondary necrosis. In support of this, Cunha and colleagues demonstrated that defects in LAP restricts tumor immunosuppression afforded by efferocytosis (13). Hence, these studies provide evidence that LAP may also be an influential contributor to the production of tolerogenic signals that are activated upon engulfment of apoptotic cells during efferocytosis. Martinez et al. further showed that in the absence of LAP, phagocytosis of apoptotic bodies still occurs but the vesicles remain in the phagocyte for a prolonged duration or are not digested, and points to a critical role of LAP in finalizing the intracellular digestion process (12). Hence, in the context of cancer, defects in LAP have paradoxically beneficial effects including the activation of tumor infiltrating lymphocytes (TIL), enhanced effector T cells activity and elevated secretion of inflammatory mediators including stimulator of interferon genes (STING), type I interferons (IFNs) and tumor necrosis factor alpha (TNF-α) (13). Taken together, these data suggest that, (1) LAP is critical for governing the digestion of the engulfed cellular debris and prevention of inflammatory and immune responses, (2) internalization of dead or dying cells alone is not solely responsible for immunosuppressive activities in the context of clearing apoptotic cells, and (3) LAP activation plays a critical role in immune tolerance. These recent findings of LAP as a concomitant effector of efferocytosis brings new significance to the processes that govern the clearance of apoptotic cells in the tumor microenvironment (TME), where there is frequent instances of programmed cell death. To that end, we discuss recent findings for the LAP signaling network as another mechanism that is hijacked by tumor cells to promote their growth and aggressiveness.

## Overview and Molecular Mechanism of Lap During Phagocytosis of Apoptotic Cells

**Figure 1:**
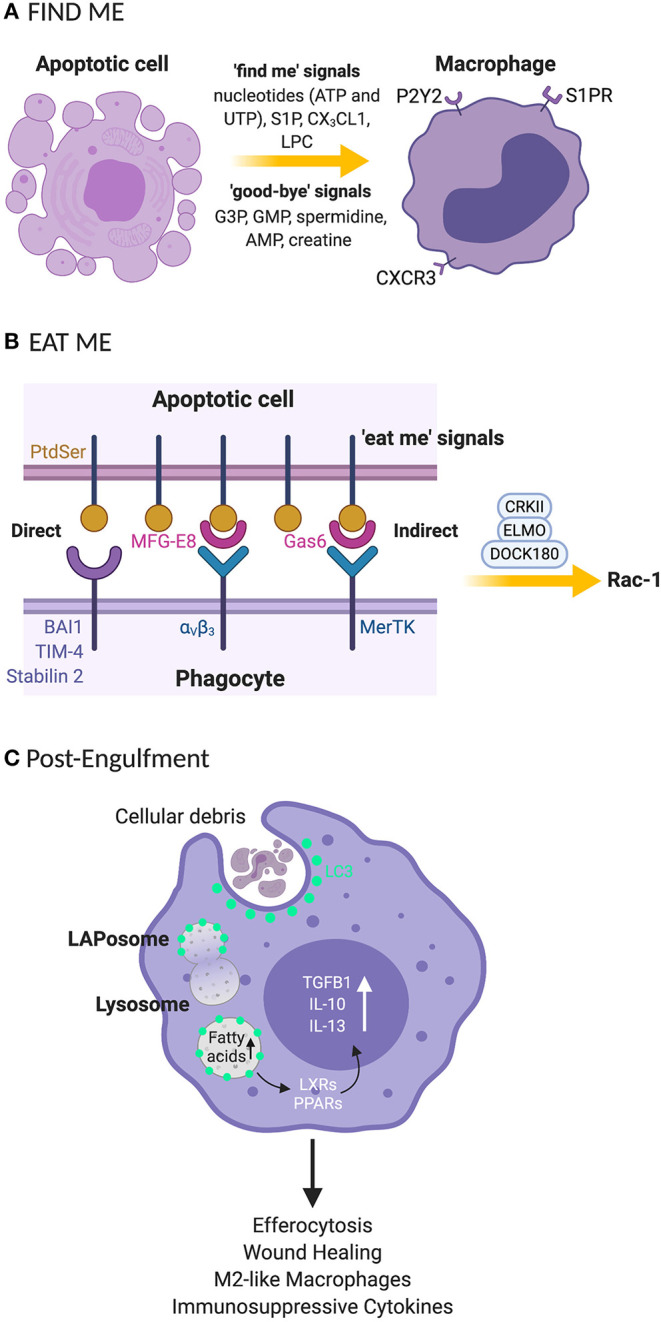
Clearance processes for apoptotic cell by phagocytes rely on “find me” and “eat me” signals that signal their internalization **(A)** “Find me” signal: apoptotic cells release signals that attract phagocytes to the site of programmed cell death. These signals include nucleotides (UTP and ATP), S1P, CX3CL1, and LPC. Phagocytes recognize the “find me” signals using cognate receptors such as P2Y2, S1PRs, CXCR3, and G2A. **(B)** “Eat me” signal: the apoptotic cells express “eat me” signals that allow phagocytes to recruit surface receptors and bridging molecules to identify and engulf apoptotic cells. PtdSer is a primary “eat me” signal expressed by phagocytes and is directly recognized by phagocytic receptors including BAI, TIM4, and stabilin 2. Phagocytes can employ avB3 and tyrosine kinase receptor such as Mertk bind to PtdSer indirectly through bridging molecules such as MFG-E8 and Gas-6, respectively. **(C)** The engulfment process: after recruitment of engulfment receptors via the CRKII-ELMO-DOCK180 complex within phagocytes, rac-1 signaling pathway is activated for phagosome formation. Once the phagosome is formed, LC3 is recruited to phagosomes (now the LAPosome) leading to lysosome-mediated digestion of the internalized apoptotic body. Degraded products release fatty acids that stimulate LXR and PPARγ for cholesterol efflux leading to the production of anti-inflammatory cytokines such as TGF-β, IL-10 and IL-13. UTP, uridine 5′ triphosphate; ATP, adenosine 5′ triphosphate; S1P, sphingosine-1-phosphate; CX3CL1, C-X3-C Motif Chemokine Ligand 1; LPC, lysophosphatidylcholine; P2Y2, purinergic receptors; SIPRs, sphingosine-1-phosphate receptors; CXCR3, C-X-C motif chemokine receptor 3; G2A, G-protein-coupled receptor; PtdSer, phosphatidylserine; BAI, brain-specific angiogenesis inhibitor 1; TIM-4, T cell immunoglobulin mucin receptor-4; avB3, alpha-v beta-3; mertk, mer proto-oncogene, tyrosine kinase; MFG-E8, milk fat globule-EGF factor 8 protein; Gas-6, growth arrest-specific 6; ELMO, engulfment and cell motility protein; DOCK, dedicator of cytokinesis; CRKII, chicken tumor virus no. 10 (CT10) regulator of kinase II; G3P, glycerol-3-phosphate guanosine; GMP, 5′-monophosphate; AMP, adenosine monophosphate; LC3, microtubule-associated protein 1A/1B-light chain 3; LXR, liver X receptor; PPARγ, peroxisome proliferator-activated receptor gamma; TGF-β, transforming growth beta; IL-10, interleukin 10; IL-13, interleukin-13. *Created with BioRender.

**Figure 2:**
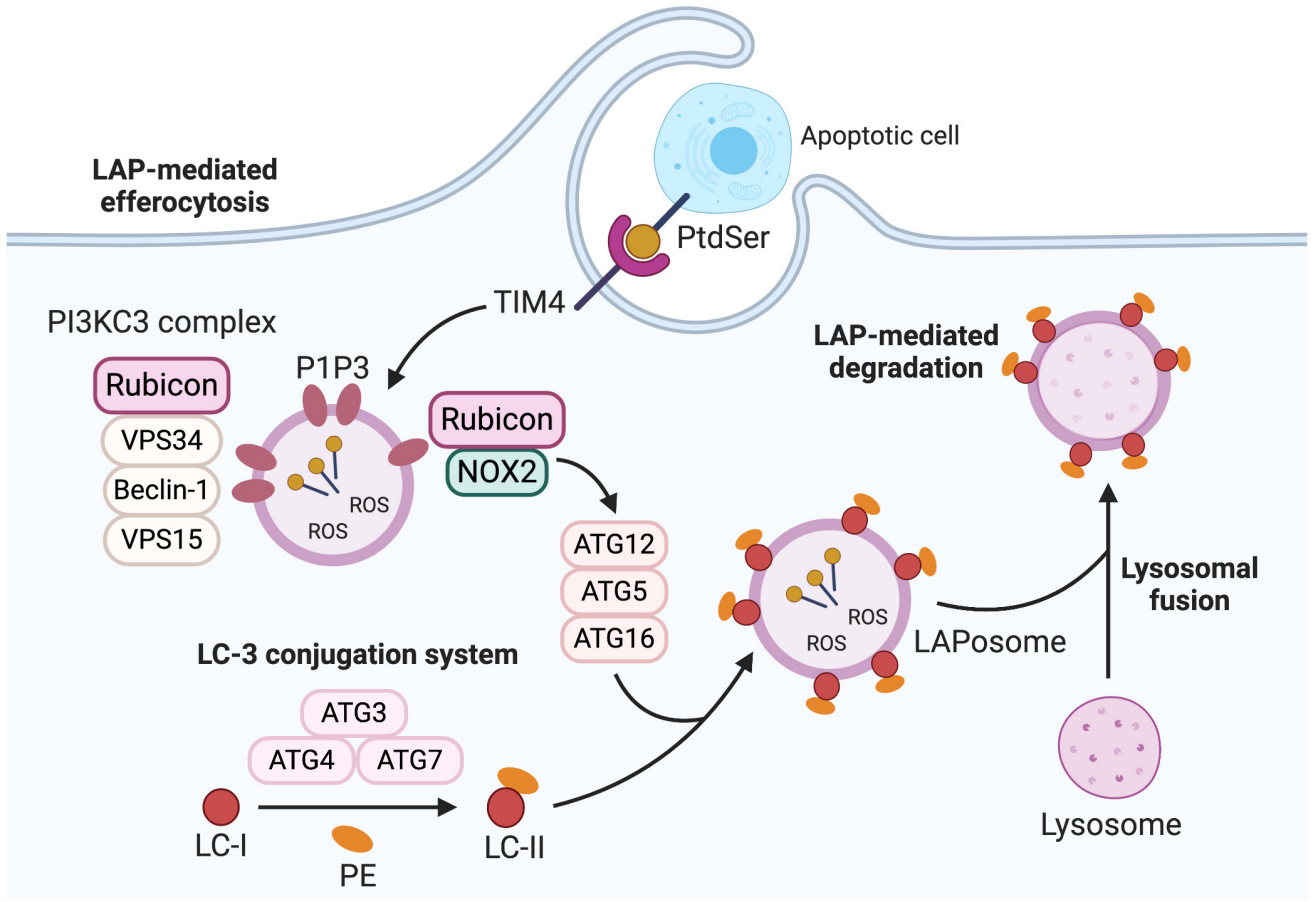
LC3-Associated phagocytosis (LAP). LAP recruitment process is triggered for degradation of the phagocytosed cargo following phagosome formation. The cargo is recognized by cell recognition receptors such as TIM-4 which causes the cargo to be engulfed in a single membrane phagosome called LAPosome. This process is initiated by recruitment of PI3KC3 complex consisting of Rubicon, vps34, beclin-1, and vps15 which enable PI3P to be localized to the LAPosomes. This stabilizes the NOX2 complex to produce ROS which is necessary to recruit LC3-II to the phagosome (LAPosome). The LAPosome then fuses with lysosomes to mature and to effective e digest the cargo. TIM-4, T-cell immunoglobulin and mucin domain family of receptors; PI3KC3 complex, Phosphatidylinositol 3-kinase catalytic subunit type 3; Rubicon, Run domain beclin-1-interacting and cysteine-rich Domain-containing protein; VPS34, Vacuolar protein sorting 34; VPS15, vacuolar protein sorting 15; PI3P, phosphatidylinositol 3-phosphate, NOX2, NADPH oxidase 2; ROS, reactive oxygen species; LC3-II, microtubule-associated protein 1A/1B-light chain 3-II. *Created with BioRender.

Apoptotic cells are cleared in a tightly controlled and coordinated manner, to avoid damaging healthy tissues. The removal processes can be partitioned into four functional stages: (1) The release of chemoattractant “find me” signals (14) and “good-bye” metabolites from cells undergoing programmed cell death. “Find me” signals such as nucleotides (adenosine triphosphate; ATP and uridine triphosphate; UTP), sphingosine-1-phosphate (S1P), CX3C motif chemokine ligand 1 (CX3CL1 or fractalkine) and lysophosphatidylcholine (LPC) and “good-bye” metabolites including glycerol-3-phosphate (G3P), guanosine 5′-monophosphate (GMP), spermidine, adenosine monophosphate (AMP) and creatine (14) are released as demonstrated in Figure 1A. This secretory activity promotes the recruitment of phagocytes or neighboring cells to the area of cell death in the tissue compartment. (2) The expression of “eat me” signals on the surface of apoptotic cells regulates the recognition by the phagocyte, subsequent engulfment of the cellular debris and preparatory steps in the mechanisms governing phagosome formation (Figure 1B). (3) Recruitment of the LC3 conjugation system (Figure 1C) to the phagosome including autophagy related proteins and factors (Atg)12-Atg5-Atg16L1, Atg3, Atg7, NADPH oxidase 2 (NOX2), reactive oxygen species (ROS), Phosphatidylinositol 3-kinase catalytic subunit type 3 (PI3KC3 complex) comprising Beclin-1, Vacuolar protein sorting 34 (VPS34), and UV radiation resistance associated (UVRAG) and Rubicon (RUN domain Beclin-1-interacting and cysteine-rich domain-containing protein) for LAPosome formation (Figure 2) where the engulfed cellular debris can be digested. (4) Post-digestion activities (Figure 1C) including the release of inflammation-resolving cytokines, immune tolerogenic signals, and exportation of breakdown products into the cytosol for metabolic recycling that service intracellular biosynthetic pathways (15–18). This previously under-recognized phenomenon for efferocytosis; localization of LC3 to phagosome, is now understood to be an essential step for lysosomal trafficking and selective immunologically silent removal of apoptotic cells (9, 12).

Finite steps involved in LAP are now being identified (Figures 1B, 2). LAP is initiated upon engulfment of apoptotic cells that have externalized eat me signals such as phosphatidylserine (PtdSer) which binds to T-cell immunoglobulin and mucin domain family of receptors (TIM); TIM-1, TIM-3, and TIM-4 or stabilin-1, stabilin-2, and the GPCR brain angiogenesis inhibitor 1 (BAI1). Alternatively, phagocytes can employ tyrosine kinase receptors (TYRO3, AXL, and Mer) also known as TAM receptors to bind to PtdSer indirectly through bridging molecules such as Gas-6 and Protein S. Interaction via these receptors stimulates the CRKII-ELMO-DOCK180 complex within phagocytes to activate the rac-1 signaling pathway (Figure 1B) (19–22). This leads to cytoskeletal rearrangement and internalization of the apoptotic cell resulting in phagosome formation. Once internalized, the phagosome recruits Rubicon which facilitates the activity of a Class III PI3K complex containing UVRAG, but which lacks Atg14 and Ambra 1 (used in canonical autophagy) (23). Consequently, phosphatidylinositol 3-phosphate (PI3P) is generated on the fully formed and sealed phagosome during LAP (10, 24). The timing of PI3P generation during LAP is different from that of canonical autophagy where PI3P is generated in some portions of autophagophore for closure and autophagosome cup formation (11, 25). This process is necessary to stabilize the NOX2 complex, and thereby sustains the production of ROS which is crucial for recruiting the LC3 conjugation system components such as Atg5, Atg3, Atg12, Atg7, and Atg16. This leads to LC3 lipidation and its localization to the phagosome, as a necessary prerequisite for the formation of the LAPosome and to promote the fusion of lysosomes (Figure 2). The phagosomal content can then be efficiently processed via enzymatic digestion, and hence the immune response is better regulated to protect against autoimmunity and inflammation.

Rubicon plays a critical role as a mediator of LAP. Rubicon (via its binding to NOX2) favors the initiation of LAP-associated non-canonical autophagic activities over canonical autophagy (26), by reducing VPS34 lipid kinase activity, which also promotes the production of PI3P on LAPosomes rather than autophagosome (27–29). Hence, further requisites for LAP is NOX2 and the production of ROS. The NOX2 subunit p40phox binds to PI3P, effectively sequestering this factor as a consequence of Rubicon activity. If ROS production is dysregulated and p40phox subunit of NOX2 fails to associate with LAPosome, leading to LAP impairment (23). Hence, Rubicon-dependent production of PI3P is integral for these processes. Further to this, Yang et al. recently showed that Rubicon directly associates with the p22phox and gp91phox subunits of NOX2 to stabilize the complex for optimum and continuous production of ROS (30). Cells or mice altered to lack Rubicon exhibit a destabilized NOX2 which thereby inhibits ROS necessary for LAP. The consequent of this is inefficient recruitment of essential LAP-related factors including ATG5, ATG7, and LC3-II to the membrane of phagosome. Moreover, cells deficient in NOX2 exhibit LAPosomes that incorporate PI3P, and exogenous induction of superoxides that serve to enhance LC3-II recruitment that initiates canonical autophagy instead of LAP (23, 29, 31). Hence, the proper regulation and expression of Rubicon is an integral part of LAP (i.e., PI3P produced via Rubicon-dependent pathways), and the interaction of Rubicon with NOX2 is a necessary event for this mode of non-canonical autophagy. As a result, Rubicon represents a new and attractive therapeutic target to specifically modulate LAP (vs. canonical autophagy) in diseases such as cancer. Considering that Rubicon and/or LAP forms a critical component of efferocytosis, we next highlight correlative studies between tumor progression and efferocytosis.

## Efferocytosis in the TME Promotes Tumor Progression and Metastatic Potential

Women diagnosed with breast cancer within 5 years of postpartum (which is characterized by massive cell death) have higher mortality rates, compared with nulliparous women (where cell death is relatively low) diagnosed with breast cancer (32–34). Additionally, breast cancer cells transplanted into involuting postpartum mammary glands grow and invade more rapidly than do cells in the mammary glands of a nulliparous host, even when corrected for age and histological grade (35, 36). This raises the possibility that conditions in the postpartum breast increase the aggressiveness of established tumors. Recently, massive cell death and the subsequent efferocytosis in postpartum involution have been identified as factors that are associated with the pro-tumoral and metastatic features of breast cancers in parous women (37). This is based on the observation that increased levels of efferocytosis occurs during postpartum involution and facilitates influx of wound-healing macrophages that are supportive of tumor progression and metastasis (37, 38).

Postpartum involution links the growth and metastasis of tumors to efferocytosis (37). This is consistent with the observation that efferocytosis receptors TIM-4 and MerTK are elevated in various cancer cells which is reported to correlate with disease severity (39, 40). In addition, a scenario in which cancer cells express MFG-E8 (41), NOX2 (42), PtdSer (21), in proximity to M2 macrophages (43) correlates with tumor aggressiveness. These observations suggest that phagocyte-homing in response to debris generated by tumor cells contributes to tumor-cell proliferation and may be heightened by the cytotoxic effects of anti-tumor therapies (44). Further, the promotion of tumor cell death by radiotherapy and chemotherapy can also lead to increased expression of efferocytic receptors such as Mertk (45) and TIM-4 (46). These findings suggest that targeting efferocytosis activity in the surrounding tumor microenvironment may mitigate this phenomenon and heighten the efficacy of conventional anti-tumor therapies.

Efferocytosis of dead or dying tumor cells is a rapid process involving engulfment receptors required for efficient clearance of the cellular debris. Several studies have shown that blocking the incorporation of apoptotic cells by inhibiting engulfment receptors such as TIM-4, MFG-E8, MerTK and its ligands including Gas6 and protein S, is an effective strategy for the treatment of tumors (41, 47–49). An emerging area of study is the identification of targets critical for the degradation process of engulfed cargoes during efferocytosis. Internalization of apoptotic tumor cells *per se*, of course, does not influence tumor progression. Immune tolerogenic signals released by the digested apoptotic cells also contribute to a mechanism whereby tumors are able to evade clearance by lymphocytes and monocytes (13). LAP is a key mechanism involved in the final stages of clearing apoptotic tumor cells (13). LAP activity in dendritic cells and macrophages effectively clears cellular debris leading to the secretion of anti-inflammatory and immunosuppressive mediators (12, 50). Moreover, elevated expression of efferocytosis receptors by tumor-derived apoptotic cells, in the absence of LAP, can enhance tumor-progression. Hence, a more complete understanding of LAP in the phagocytes that home to the tumor microenvironment may offer new strategies for the management of cancer.

## The Role of Lap in Tumor Progression

During efferocytosis, LAP facilitates the fusion of phagosome to lysosomes to enhance hydrolytic digestion and elimination of apoptotic cell constituents (23). Once apoptotic cells are digested in phagolysosomes, phagocytes become burdened with macromolecular digest components that they then either use in biosynthetic processes or efflux to the extracellular environment. An overload of cholesterol from degraded apoptotic cells stimulates members of the peroxisome proliferator-activated receptor gamma (PPARγ) and liver X receptor (LXR) families of nuclear receptors as illustrated in Figure 1. These nuclear receptors inhibit pro-inflammatory cytokines and drive the polarization of M2-like phenotype which can mediate the production of anti-inflammatory cytokines. This is one important mechanism that reduces inflammatory signals following the removal apoptotic cells (51, 52), with immunogenic consequences including the suppression of tumor immune responses and increased resistance to immunotherapies.

**Figure 3:**
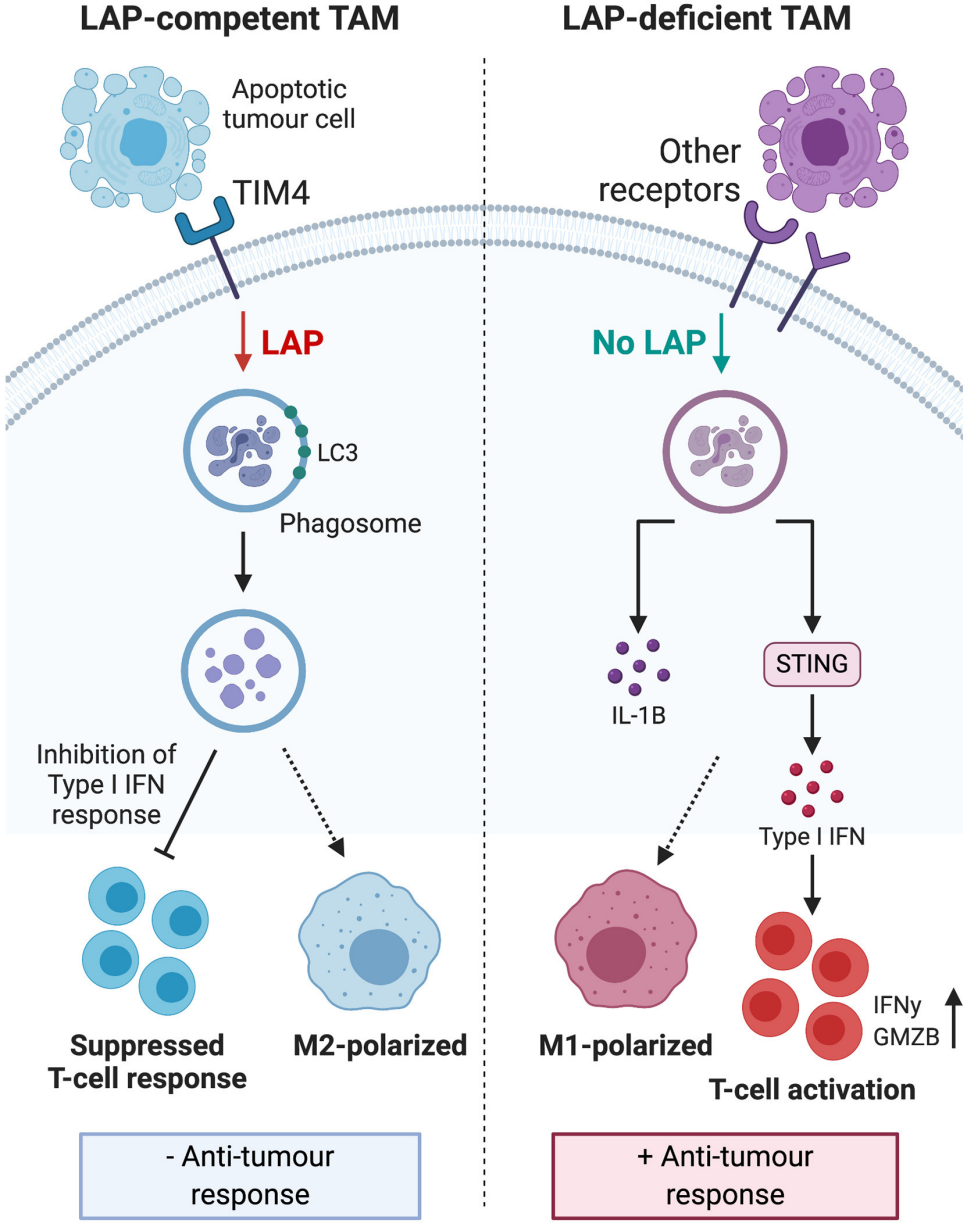
Engulfment of apoptotic tumor cells induces LAP to inhibit type I IFN response and polarize tumor associated macrophages toward the immunosuppressive M2 phenotype. This process leads to suppressed TIL function, reduced anti-tumor response and ultimately, sustained tumor survival. LAP deficiency leads to polarization of TAMs toward the pro-inflammatory M1 phenotype and stimulation of a STING-dependent type I IFN response, which enhances TIL function and expression of IFNγ to inhibit tumor growth. Taken together, these mechanisms suggest that targeting the LAP pathway may have therapeutic potential. IFN, interferon; TIL, tumor-infiltrating lymphocytes; STING, stimulator of interferon genes; IFNγ, interferon gamma. *Created with BioRender.

LAP activity in dendritic cells and macrophages enhances phagocytic removal of pathogens and cells undergoing programmed cell death (9, 50). This is evidenced in LAP-deficient dendritic cells that exhibit impaired efferocytosis and elevated expression of major histocompatibility complex class I (50) that is needed for antigen presentation and anti-tumor immunity (50, 53). Moreover, macrophages and mice altered to obviate LAP, internalize and accumulate undigested apoptotic cells. These cells exhibit STING-dependent IFN responses, M1 polarization, pro-inflammatory mediators, granzyme B and enhanced anti-tumor immune response (11, 13, 54, 55). Further, DNA from apoptotic cells can activate STING and mediate immune recognition of tumor cells (56) which can also lead to interferonopathy and autoimmunity (57). This suggests that while LAP-dependent degradation of the DNA of engulfed apoptotic tumor cells counters the induction of inflammation and autoimmunity, it can also suppress anti-tumor immune responses. Hence, it follows that internalization of apoptotic material *per se*, does not lead to M2 polarization and immunosuppression. Rather it is the digested and degraded products of apoptotic cells that induce immune tolerogenic signals utilized by tumor cells to promote their growth and progression during efferocytosis. As illustrated in Figure 3, LAP facilitates digestion and removal of apoptotic tumor cells upon phagosomal engulfment that has unfortunate consequence of switching anti-tumor M1 macrophage phenotype to a pro-tumor M2 phenotype, leading to tumor progression and severity (13). This indicates that LAP participates in the regulatory events that form a complex network of interactions that collectively enhance anti-inflammatory and pro-tumorigenic effects of apoptotic cells and efferocytosis. Therefore, targeting LAP provides the attractive possibility of attenuating tumor progression and potentially serves as a target that could be therapeutically modulated to help prevent tumor aggressiveness.

## Lap Contributes to Tumor Progression by Promoting M2 Macrophage Polarization

Macrophages are immune cells that are widespread throughout the body. They exhibit diverse functions including the regulation of inflammation, homeostasis, and tumor immunity. They are generally categorized into classically activated or M1 macrophages and alternatively activated or M2 macrophages. M1 macrophages are activated by Th1 lymphocytes, interferon-γ (IFN-γ), TNF-α, and natural killer cells (NK), while M2 macrophages are induced by cytokine signatures presented by Th2 cells such as interleukin-13 (IL-13), IL-10, and IL-4. Tumor-associated macrophages (TAMs) are usually triggered by factors that polarize macrophages to M2 phenotype and are often infiltrated into tumor sites to suppress the cytotoxic function of anti-tumor immune cells (43, 58, 59). This leads to dysregulation of tumor immune response contributing to the immunosuppressive tumor microenvironment. Indeed, observations from *in vitro* studies and clinical findings from cancer patients show that increased TAM infiltration correlates with poor prognosis (43). Accordingly, mitigating TAM infiltration, by blocking M2 activation or stimulating a pro-inflammatory M1 phenotype in TAMs, reduces tumor growth and metastasis (59–61).

**Figure 4:**
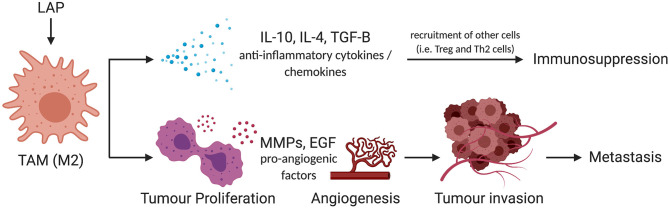
Schema of mechanism by which LAP could facilitate metastatic potential of tumors via M2 polarization. M2 promotes tumor progression through IL-10, IL-4, and TGF-β-mediated immunosuppression. They also trigger pro-survival signals such as MMPs and EGF to amplify metastatic potential of tumors by facilitating the invasion and spread of cancer cells to distant sites within the body. IL-10, interleukin-10; IL-4, interleukin-4; TGF-β, transforming growth factor-beta; MMP, matrix metallopeptidases; EGF, epidermal growth factor (74). *Created with BioRender.

Importantly, M2 macrophages promote tumor aggressiveness and progression. This phenomenon is exhibited through three mechanisms as shown in Figure 4. Firstly, rapidly proliferating tumor cells that outpace the rate of blood supply need to establish new blood vessels through a process known as angiogenesis. M2 macrophages produce growth factors such as Epidermal Growth Factor (EGF), Fibroblast Growth Factor (FGF) to promote vascularization and allow highly proliferative tumors to obtain adequate blood supply (62–65). This enhances metastatic spread as it provides the principal route by which tumor cells exit the primary tumor site and enter the circulation. Secondly, secretion of matrix metalloproteases by tumor-infiltrating M2 macrophages has been shown to contribute to the degradation of the Extra Cellular Matrix (ECM) which facilitates the invasion and spread of cancer cells to distant sites within the body and secondary metastasis (66). This phenomenon has been effectively described in human tongue, squamous cell, and colorectal cancer (67, 68). Thirdly, the production of anti-inflammatory cytokines and chemokines can also shift the immune response toward an immunotolerogenic phenotype, which is unable to sustain cell-mediated tumor immunity (69–71). This antagonizes activities of effector T cells and enhances tumor growth and survival, as they are spared from immune destruction. For these reasons, inhibition of M2 macrophages has shown therapeutic efficacy in the management of several tumors (59, 72, 73), and provides evidence that the characterization of factors that fine-tune M2 macrophage polarization can provide novel therapeutic options for promoting tumor immunity.

LAP has been identified as a new mechanism that participates in M2 polarization, and promotes an immunosuppressive environment that favors the tumor growth (13, 61, 75). Therefore, the involvement of LAP in macrophage function warrants further elucidation. This is evidenced by pro-tumor events that are established by LAP related to macrophage function. For example, (1) markers of tolerance and the polarization of macrophages to M2 phenotype is observed in LAP-sufficient tumor animal models (13, 76–78), (2) the M2 phenotype correlates with poor prognosis and promotes tumor progression (2, 79), and (3) LAP-sufficiency in tumor mouse models has been shown to accumulate M2 macrophages that support the pro-tumorigenic effects of TAMs (13). Therefore, M2 macrophage accumulation in tumors after chemotherapeutic treatment (80, 81) may be linked to LAP when it is called to enhance the efferocytosis of the apoptotic tumor cells. This causes the reduction of cytotoxic T cell activity to limit tumor immune responses and enhance the cancer cell proliferation (13, 82). Further, Cunha and colleagues showed that genetic or pharmacological inhibition of LAP overcomes some of the pro-tumor effects of TAMs e.g., by increasing CD8+ T cells function and reducing M2 macrophage production (13). These observations point toward an unrecognized target to combat the progression of tumors.

## Characterization of Lap as A Distinct Pathway Reveals A New Therapeutic Approach to Control Tumor Growth

A current aim for cancer therapy is to target autophagy in the tumor microenvironment (83, 84). For example, the translocation of LC3-II to membranous structures, considered to be a hallmark for autophagy, has been associated with tumor progression (85). Further, inhibition of LC3 recruitment can potentiate anti-tumor immune response and reduce tumor growth (86, 87). Recent discovery that LC3 is recruited to phagosome membranes via LAP provides evidence that at least some pro-tumor functions of LC3 recruitment may be specifically linked to LAP (10, 13). Indeed, pro-tumoral qualities of some autophagy components have been associated with LC3 recruitment to phagosome membranes via LAP. Noteworthy, translocation of LC3 to phagosomes is a reliable marker for LAP, that distinguishes it from processes related to canonical autophagy (23). Also, while LAP involves the recruitment of LC3 to single phagosome membranes (10, 88), autophagy utilizes LC3-enriched double membrane autophagosomes (89). Hence, delineating between these two processes can be used to identify unrecognized roles and contribution of LAP in tumor progression.

A critical distinction between LAP and autophagy is that while LAP requires Beclin-1, PI3KC3 complex, Atg5 and Atg7 for recruitment of LC3 to membranous structures, unlike autophagy, LAP does not require Unc51-like kinase 1 complex (ULK-1) or FAK family–interacting protein of 200 kDa (FIP200) (90–93). Moreover, while both LAP and autophagy require mediators such as SLAM (signaling lymphocyte-activation molecule) receptors to interact with the PI3KC3 complex to regulate phagosome and autophagosome maturation, respectively, unlike autophagy, LAP employs Rubicon-containing PI3KC3 and does not require Atg14 or ULK-1 to mediate LC3 recruitment process (23, 26). In fact, inhibiting Rubicon specifically block LAP without inhibiting canonical autophagy process.

Moreover, even though VPS34 activity is required for both autophagy and LAP, VPS34 produced in the absence of Rubicon triggers autophagy instead of LAP (23, 31). Conversely, Rubicon swaps VPS34 activity on autophagosome for LAPosme (23, 26, 94). This again characterizes Rubicon as an indispensable and specific component of LAP. These characterisations of LAP as a distinct mechanism provides a new avenue to regulate tumor growth without interfering with canonical autophagy processes.

## Targeting Core Components of Lap in Tumors

**Figure 5:**
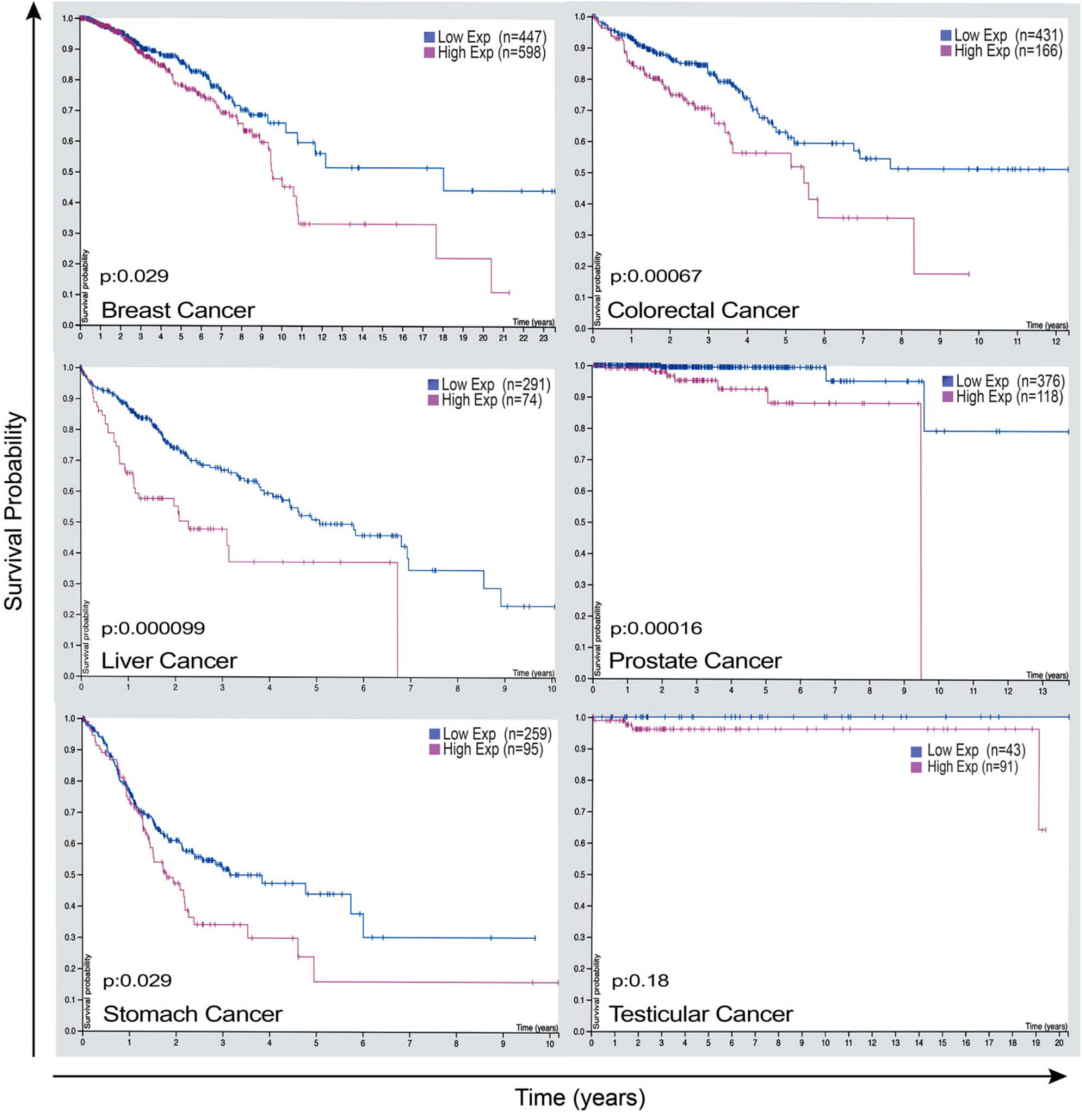
Rubicon expression as a potential prognostic marker that has the power to predict survival outcome of some cancer patients. Representative images of Rubicon expression and survival rate of patients with breast cancer, colorectal cancer, liver cancer, prostate cancer, stomach cancer, and testicular cancer. Cancer patients overexpressing Rubicon have lower survival rate compared with patients with lower Rubicon expression. This demonstrates the need for further studies to establish prognostic values of Rubicon in different types of cancers. Credit: Human Protein Atlas, www.proteinatlas.org/humancell (95). Image available at the following URL: v19.proteinatlas.org/humancell.

Given that LAP elicits pro-tumor effects in the context of cancer, cancer cells expression of factors that lead to these phenomena may have significant therapeutic value. The expression of Rubicon is elevated in cancer and is strikingly associated with poor prognosis in patients with breast cancer, endometrial cancer, testicular cancer, liver cancer, colorectal cancer, and stomach cancer as shown in Figure 5 (95). This clear link between the abundance of Rubicon and poor prognosis of cancer (Figure 5), calls for a better understanding of the events associated with the pro-tumor effects of Rubicon to inform prognostic and therapeutic investigations. What is certain is that Rubicon is associated with LAP and is a requisite member of the LAP pathway (27). Further, Rubicon is upregulated following internalization of apoptotic cells to recruit LC3-conjugation system for LAP initiation and subsequent degradation of apoptotic cells (23). This supports the view that Rubicon expression forms a critical component of the processes required to remove apoptotic cells. In fact, inhibition of Rubicon in preclinical settings enhances immune activation and restricts tumor growth by ablating LAP-mediated apoptotic cell removal. Conversely, Rubicon expression increases tumor progression and limits immune responses through LAP induction (13). This suggests that LAP may be an influential pathway through which Rubicon mediates tumor progression and decreases survival rate in some cancer patients.

**Figure 6:**
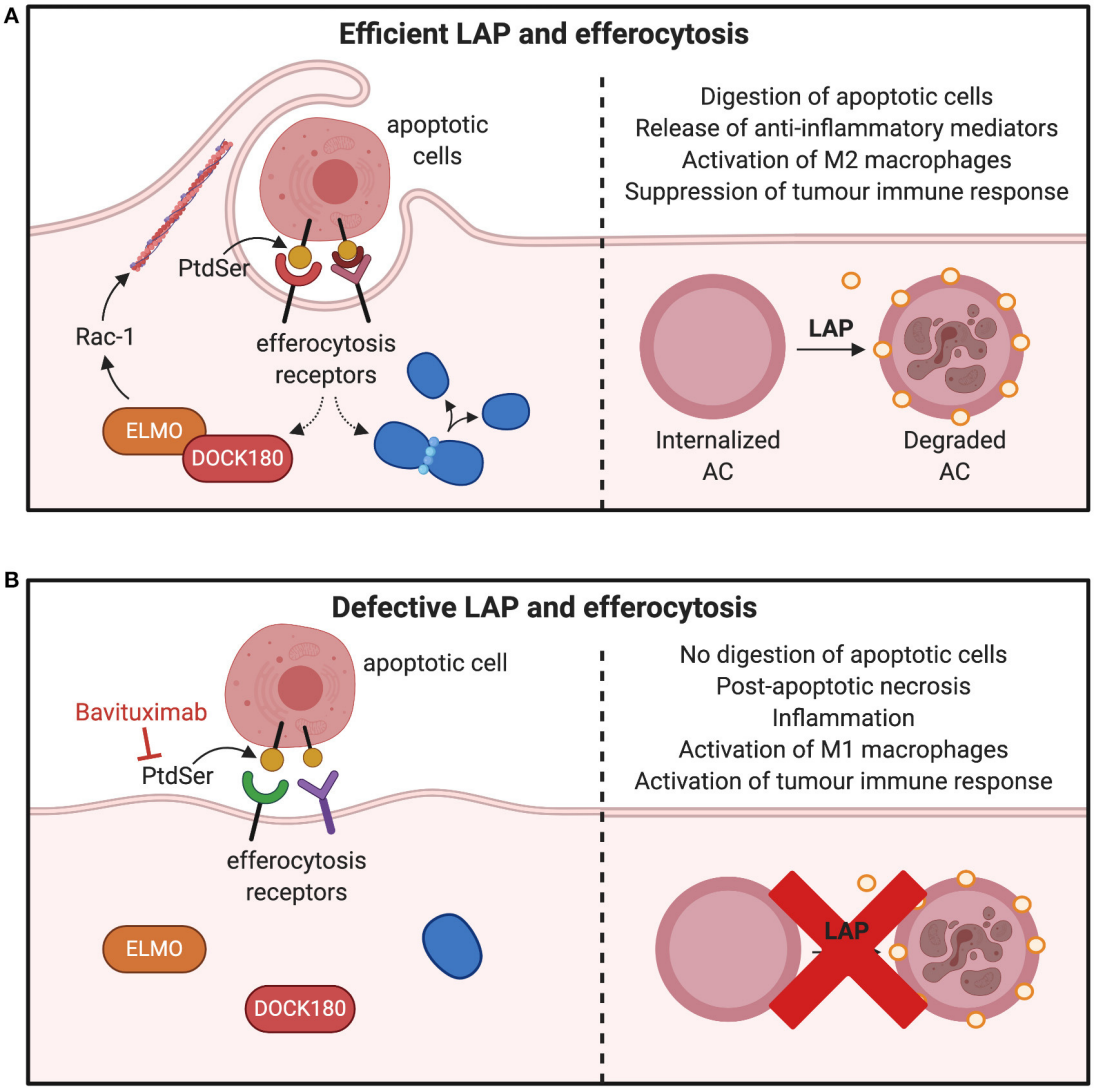
Consequences of inhibiting factors involved in efferocytosis and LAP. **(A)** Macrophages interact with PtdSer externalized on apoptotic cells through efferocytic receptors on the surface of phagocytes. Many PtdSer receptors stimulate interactions to activate Rac1 and cytoskeletal arrangement for phagosome formation and internalization. Once apoptotic cells are internalized in the phagosome, LC3-recruitment process through LAP is triggered to fuse LC3 to phagosomes membranes, which facilitates phagolysosomal fusion and subsequent digestion apoptotic cell. This results in production of M2 macrophages and suppression of tumor immune activation. **(B)** Inhibition of PtdSer leads to defects in the phagosome formation by preventing the cytoskeletal rearrangement through Rac-1 and ELMO–DOCK180 interactions. This leads to inefficient clearance of apoptotic cells and polarization of M1 macrophages to release mediators that potentiate tumor immune responses. PtdSer, phosphatidylserine; LC3, microtubule-associated protein 1A/1B-light chain 3-II; ELMO, engulfment and cell motility protein; DOCK, dedicator of cytokinesis. *Created with BioRender.

PtdSer is another integral component of LAP induction and efferocytosis (9, 96). It is usually expressed on the surface of apoptotic cells to allow phagocytes to remove the dead or dying cells through efferocytosis and LAP (21, 97). It can be predicted that its inhibition may ablate LAP and therefore many pro-tumorigenic consequences as shown in Figure 6. For example, pre-clinical models of B16F10 melanoma demonstrates that inhibiting PtdSer receptor, TIM-4 is an effective approach to reduce LAP and tumor growth (13). Indeed, expression of PtdSer on the surface of apoptotic cell is an early event during apoptosis and is markedly increased during chemotherapeutic treatment of tumors (98). PtdSer is also overexpressed in different cancer types such as glioblastoma, breast cancer and astrocytoma (99, 100). Its inhibition could lead to LAP impairment and subsequently result in a decrease in M2-like tumor associated macrophages and a switch in the expression of immunosuppressive cytokines to immunostimulatory cytokines (98). Indeed, Bavituximab which blocks PtdSer is providing beneficial outcomes for some cancers in phase II clinical trials (101, 102).

Moreover, several clinical studies are in progress to design therapies that may have the potential to prevent the impact of LAP in the tumor microenvironment. These include Blocking TGF-β signaling to inhibit the growth and metastasis of orthotopic mammary carcinoma (103). TGF-β is usually upregulated and is integral for tumor immunosuppressive effects of LAP (11, 13, 82), suggesting that targeting factors involved in the initiation of LAP as well as its resultant effects could be an effective strategy for tumor destruction.

IL-10, a further anti-inflammatory cytokine, is produced by both M2 macrophages (104) and by the tumor cells (105, 106). IL-10 can promote tumor growth and the clinical utility of inhibiting this effect is widely reported (107–109). However, the precise mechanism that informs IL-10 overexpression and its pro-tumoral qualities remains to be completely elucidated. Data from experiment settings link overexpression of IL-10 to the LAP pathway and implicate this association in LAP-mediated tumor progression (13). This underscores the role of LAP plays in IL-10 expression and provides a potential target to regulating the pro-tumor cytokine in tumor cells. Noteworthy, other biological events may also contribute to IL-10 mediated tumor progression. A better understanding of LAP as an influential driver in this regard is crucial to discover a novel approach to block the immunosuppressive and pro-tumor effects of IL-10 for effective clinical management of tumors.

V-ATPase activity has recently been shown to be required for LAP, and inhibiting V-ATPase following apoptotic cell death prevents LAP by blocking lipidation of LC3 (110, 111). This provides a further potentially novel therapeutic target for regulating LAP. Additionally, studies by Fletcher et al. suggest that WD repeat-containing C-terminal domain of ATG 16L1 inhibition arrests LAP (112). Blunting WD repeat-containing C-terminal domain (WD 40 CTD) of ATG 16L1 blocks LC3 recruitment to endolysosomal membranes during LAP without affecting canonical autophagy (112, 113).

The mode of cell death has a significant impact on the immune tolerogenic effects of LAP. Non-apoptotic cell death such as necrotic and necroptotic cells can also induce LAP to complete the efferocytosis process (9). In contrast to apoptotic cells, necrotic and necroptotic cells induce inflammatory and immunostimulatory cytokines (114, 115) and can switch macrophages from pro-tumoral M2 phenotype to anti-tumoral and immunostimulatory M1 phenotype (116, 117). This is because the extra cholesterol produced after the degradation of necrotic cells does not stimulate cholesterol efflux pathway (118) which is required for the release of anti-inflammatory cytokines essential for M2 phenotype switching (119, 120). This provides another promising approach for circumventing the pro-tumoral effects of LAP. Noteworthy, induction of non-apoptotic modalities of cell death such as necrosis or necroptosis as a new therapeutic direction to potentiate anti-tumor immunity has been suggested as a promising alternative (117, 121–123). Therefore, further studies in this regard are warranted especially as the effectiveness of non-apoptotic form of tumor cell death has not been explicitly elucidated.

## Concluding Remarks

A common effect of anticancer therapies is to induce apoptosis of cancer cells. However, there is a surprising paucity of information that considers the complex processes that are responsible for clearing the cellular debris. In line with this, there is emerging evidence that implicates the non-canonical autophagy pathway, LAP, as an essential component involved in the coordinated clearance of apoptotic cancer cells. However, unlike canonical autophagy and phagocytosis, LAP-mediated efferocytosis elicits an anti-inflammatory response in the surrounding environment that may have significant implications for the evasion of immune surveillance by cells involved in tumorigenesis. Indeed, it has been shown that mice altered to abrogate LAP as a part of the cell's clearance response exhibit a reduction in tumor growth, while the restoration of LAP influences tumor progression. Further, cancer tissues overexpression of Rubicon predicts adverse overall survival in many cancer patients (95). These findings have several clinical implications: (1) Rubicon expression in tumor tissues comprising infiltrated TAMs and other phagocytic cells can lead to modulation of LAP pathway in the TME (13). This implies that LAP can specifically be targeted in the TME to restrict tumor growth. (2) Rubicon expression could be a prognostic marker in different types of cancers and (3) Therapies that could inhibit Rubicon expression could be incorporated into clinical trials as drugs that regulate LAP during phagocytosis of apoptotic tumor cells. However, to the best of our knowledge, there is no drug at present that could specifically target Rubicon. Therefore, these findings point to a need to clearly determine how LAP is regulated during the efferocytosis of apoptotic tumor cells and whether alterations implicit with uncontrolled carcinogenesis favor the survival of cells that employ LAP to establish tumors of clinical importance. If so, perhaps a more immediate priority is to identify those therapeutic options which unwittingly potentiate cancer by overbalancing the recruitment of phagocytes and/or that directly promote LAP in cancer cells themselves. Given that LAP functions to regulate inflammation and undesired immune responses, inhibition of LAP while restricting tumor growth has the potential to result in autoimmunity and chronic inflammation (12). This provides a worthwhile consideration when inhibiting LAP to control tumor progression. Herein, we discuss the current understanding of LAP during the development of cancer, its relationship with efferocytosis in the tumor microenvironment, and suggest strategies that may inform current and novel therapeutic approaches.

## Author Contributions

PA drafted the manuscript and revised it critically for important intellectual content. ER, PH, HT, and CM revised manuscript critically for important intellectual content. SH revised manuscript critically for important intellectual content and have final approval for submission. All authors contributed to the article and approved the submitted version.

### Conflict of Interest

The authors declare that the research was conducted in the absence of any commercial or financial relationships that could be construed as a potential conflict of interest.
